# Phase Transition and Metallization of Orpiment by Raman Spectroscopy, Electrical Conductivity and Theoretical Calculation under High Pressure

**DOI:** 10.3390/ma12050784

**Published:** 2019-03-07

**Authors:** Kaixiang Liu, Lidong Dai, Heping Li, Haiying Hu, Linfei Yang, Chang Pu, Meiling Hong, Pengfei Liu

**Affiliations:** 1 Key Laboratory of High-Temperature and High-Pressure Study of the Earth’s Interior, Institute of Geochemistry, Chinese Academy of Sciences, Guiyang 550081, China; liukaixiang@mail.gyig.ac.cn (K.L.); hepingli_2007@hotmail.com (H.L.); huhaiying@mail.gyig.ac.cn (H.H.); yanglinfei@mail.gyig.ac.cn (L.Y.); puchang@mail.gyig.ac.cn (C.P.); hongmeilin@mail.gyig.ac.cn (M.H.); 2 University of Chinese Academy of Sciences, Beijing 100039, China; 3 State Key Laboratory of Structural Chemistry, Fujian Institute of Research on the Structure of Matter, Chinese Academy of Sciences, Fuzhou 350002, China; liupengfei20170208@163.com

**Keywords:** high pressure, diamond anvil cell, Raman spectroscopy, electrical conductivity, phase transition, pressure-induced metallization

## Abstract

The structural, vibrational, and electronic characteristics in orpiment were performed in the diamond anvil cell (DAC), combined with a series of experimental and theoretical research, including Raman spectroscopy, impedance spectroscopy, atomic force microscopy (AFM), high-resolution transmission electron microscopy (HRTEM), and first-principles theoretical calculations. The isostructural phase transition at ~25.0 GPa was manifested as noticeable changes in the compressibility, bond lengths, and slope of the conductivity, as well as in a continuous change in the pressure dependence of the unit cell volume. Furthermore, a pressure-induced metallization occurred at ~42.0 GPa, accompanied by reversible electrical conductivity. We also determined the metallicity of orpiment at 45.0 GPa by first-principles theoretical calculations, and the results were in good agreement with the results of the temperature-dependent conductivity measurements. The HRTEM and AFM images of the recovered sample confirmed that orpiment remains in the crystalline phase with an intact layered structure and available crystal-shaped clusters. These high-pressure behaviors of orpiment present some crucial information on the structural phase transition, metallization, amorphization and superconductivity for the *A*_2_*B*_3_-type of engineering materials at high pressure.

## 1. Introduction

*A*_2_*B*_3_-type chalcogenides with diverse structures and physical properties could be exploited in some important industrial applications, such as thermoelectric devices, solid-state power devices, refrigerating devices, photovoltaic cells, spintronics, and quantum computation [1,2,3,4]. As a representative *A*_2_*B*_3_-type semiconductor, orpiment can be used for photocatalytic water-splitting applications [5]. The amorphous phase of orpiment is also an important material for physical applications because of its extensive use in optics and electronics [6]. Orpiment (As_2_S_3_) is a well-known binary semiconductor with optical bandgap energy (*E_g_*) of ~2.7 eV [7]. Under ambient conditions, orpiment crystallizes in a quasi-two-dimensional monoclinic structure (SG *P*2_1_/*c*, *Z* = 4), in which the layers parallel to the (010) plane are bonded by weak van der Waals forces [8].

As we know, pressure is one of the efficient methods to optimize and improve the structural and physical properties in a large number of engineering materials, such as *AB*, *AB*_2_ and *A*_2_*B*_3_ types of semiconductor compounds [9,10,11,12]. As for the representative *A*_2_*B*_3_-type compound, the pressure-induced structural phase transition, metallization, amorphization and superconductivity have already attracted considerable attention by more and more researchers in the recent several years [12,13,14,15,16]. Hence, some crucial characterizations of orpiment under high pressure and high temperature have been reported previously. Bolotina et al. [17] observed decomposition of orpiment into two high-pressure phases (AsS and AsS_2_) at a pressure above ~6 GPa and a temperature above ~800 K, by means of Xcalibur single-crystal diffraction. Besson et al. [18] found that when the pressure increased to 10 GPa at room temperature, there was no phase transition of orpiment based on optical absorption and Raman spectroscopy experiments, even though there was a decrease in the bandgap energy from 2.7 eV to 1.6 eV. Although reports about the experimental data for orpiment at pressures above 10 GPa are absent, high pressure can be applied to reduce the interatomic distances, finally resulting in metallization and a possible pressure-induced phase transition. For amorphous orpiment, from Raman spectroscopy measurements, a pressure-induced phase transition occurred at ~ 4 GPa [19,20]. In addition, the semiconductor–metal transition of amorphous orpiment has been observed at a pressure of ~45 GPa by optical reflectivity and absorption measurements [21].

The type of phase transition can be determined by the characteristic parameter variations, such as variation in the unit cell volume, crystalline lattice parameters, axial ratio and bond lengths [22,23]. These characteristic parameters can be obtained by first-principles theoretical calculations. Radescu et al. [24] recently determined that there was no phase transition in orpiment up to 16 GPa, by first-principles theoretical calculations. To verify whether orpiment undergoes a pressure-induced phase transition above 16 GPa, the characteristic crystal cell parameters should be calculated in a large pressure range.

In the present study, to systematically investigate the pressure-induced phase transition and metallization of orpiment, we determined the electrical and structural properties at pressures up to ~46.0 GPa using the DAC in conjunction with a series of experimental and theoretical methods, including Raman spectroscopy, impedance spectroscopy, atomic force microscopy (AFM), high-resolution transmission electron microscopy (HRTEM), and first-principles theoretical calculations. 

## 2. Experimental and Computational Details

### 2.1. Sample Description

**Figure 1:**
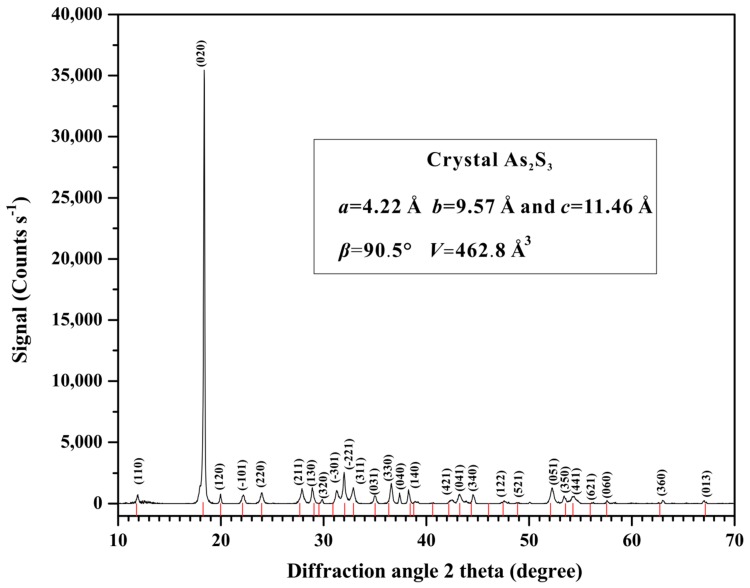
The X-ray diffraction of orpiment under ambient conditions.

In this study, the natural crystalline orpiment was gathered from Jiepaiyu ore deposit in Shimen city, Hunan province. Before the high-pressure experiments, the crystalline orpiment was crushed into a powder (~20 μm). The X-ray powder diffraction (XRD) analysis of the sample was conducted using an X’Pert Pro X-ray powder diffractometer (Phillips Company, Amsterdam, Netherlands), the Cu Kα radiation with 45 kV and 40 mA) in the State Key Laboratory of Ore Deposit Geochemistry, Institute of Geochemistry, Chinese Academy of Sciences. Figure 1 is the X-ray diffraction for orpiment under ambient conditions. The samples displayed a quasi-two-dimensional monoclinic structure (SG *P*2_1_/*c*, *Z* = 4). The data analysis and handling software JADE 6.0 was used. Some lattice constant parameters were given as follows: *a* = 4.22 Å, *b* = 9.57 Å, *c* = 11.46 Å and *β* = 90.5°. The unit cell volume (*V*) was 462.8 Å^3^.

### 2.2. High-Pressure Raman Scattering Measurements

A diamond anvil cell (DAC) with a 300 μm anvil culet was adopted in the Raman spectroscopy measurements (Renishaw, London, England) under high pressure. The pressure calibration was realized using the ruby luminescence method. Helium was used as the pressure medium to provide a hydrostatic condition, and no pressure medium was used for the nonhydrostatic condition. The Raman spectra were recorded with an Invia Raman spectrometer (Renishaw, London, England) equipped with a charge-coupled device camera (Olympus, Tokyo, Japan) and a confocal microscope (TCS SP8, Leica, Solms, Germany). The excitation laser power for the high-pressure Raman spectra measurements and fluorescence was 20 mW and 0.5–40 μW, respectively. The Raman spectra were obtained by an argon ion laser (Spectra physics; 514.5 nm, power <1 mW) in the backscattering geometry, and a Raman range of 125−425 cm^−1^ with the resolution of 1.0 cm^−1^ was employed in the process of spectral acquisition. In order to achieve a stable pressure condition, the equipment pressure stabilization time was 1 h at the predesigned pressure, before each Raman spectral measurement. The PeakFit software was employed to fit the correspondent Raman spectroscopy so as to identify the position of each Raman mode and its uncertainty. The AFM and TEM data were measured by virtue of a Multimode 8 mass spectrometer (Bruker, Karlsruhe, Germany) and a Tecnai G2 F20 S-TWIN TMP (FEI, Hillsboro, America), respectively.

### 2.3. High-Pressure Conductivity Measurements

**Figure 2:**
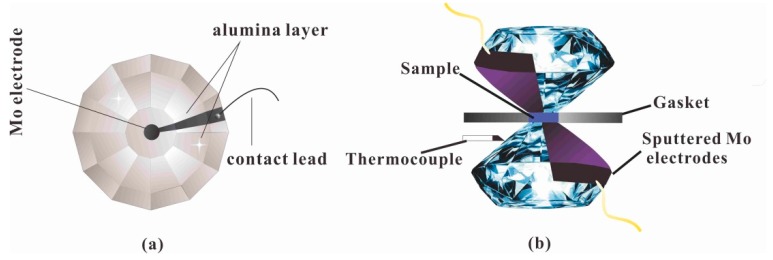
Measurement assemblage of sample for electrical conductivity at high pressure. (**a**) The structure of plate electrodes integrated on two diamond anvils. (**b**) Cross section of the diamond anvil cell (DAC) employed in the high-pressure electrical conductivity measurement.

For the high-pressure electrical conductivity experiments, a DAC with a 300 μm anvil culet was used. After pre-indented to a thickness of ~60 μm, a 180 μm hole was drilled in a T-301 gasket using a laser. Then the hole was filled with the insulating powder, which consisted of a boron nitride powder and epoxy, and another 100 μm hole was drilled as the insulating sample chamber. Figure 2 is the cross-sectional structure of the DAC. The electrical conductivity of orpiment was acquired using Solartron-1260 and Solartron-1296 impedance spectroscopy analyzers, with a frequency of 10^−1^–10^7^ Hz. For the temperature-dependent conductivity measurements, liquid nitrogen was used to obtain different temperatures. The temperature measurements were performed by a *k*-type thermocouple, which was attached to the diamond with an accuracy of 5 K. The temperature of the experimental assembly was varied by volatilization of liquid nitrogen. A similar measurement procedure and experimental assembly were presented previously [12,25,26,27].

### 2.4. Computational Details

All of the ab initio calculations were performed with the CASTEP (Materials Studio) code within the first-principles theoretical framework of density functional theory (DFT), using the pseudopotential method. The Perdew–Burke–Ernzerhof scheme in generalized gradient approximation was used to obtain the exchange and correlation terms. The Broyden–Fletcher–Goldfarb–Shanno minimization algorithm in the code was adopted to realize structural optimizations in orpiment. A cutoff energy of 360 eV was applied to the valence electronic wave functions expanded in a plane-wave basis set for monoclinic orpiment. To guarantee the high convergence in the total energy of 1 meV per atom, the special *k* points generated by 6 × 4 × 2 parameter grids for the *P*2_1_/*c* phase were acquired to achieve the integration of the Brillouin zone. All of the atoms and lattice constants were relaxed thoroughly until the force convergence reduced to less than 0.01 eV/Å, which was used to obtain the different lattice constants and atomic positions of orpiment at varied pressures. The initial structural parameters of orpiment were obtained from previously reported results [28].

## 3. Results and Discussion

**Figure 3:**
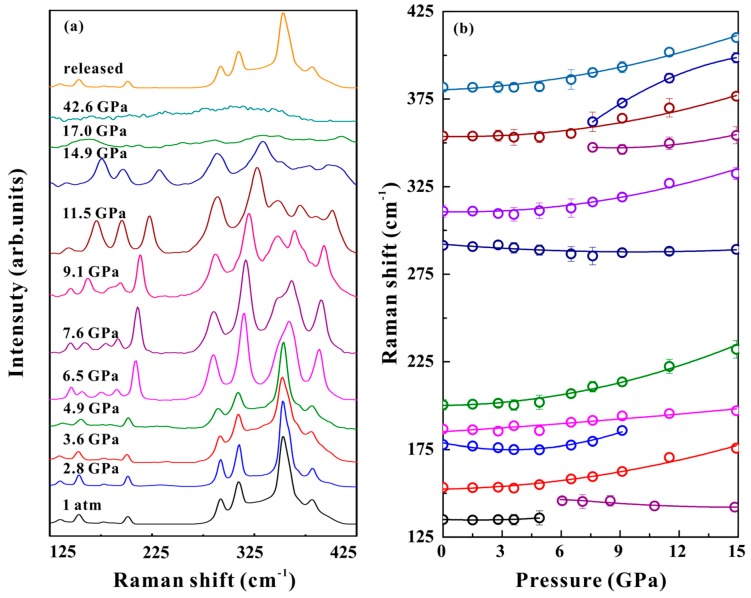
(**a**) Raman spectra of orpiment at selected pressures under non-hydrostatic condition (*λ*= 514 nm, *T*= 300 K). (**b**) Raman shift of orpiment with increasing pressure.

**Figure 4:**
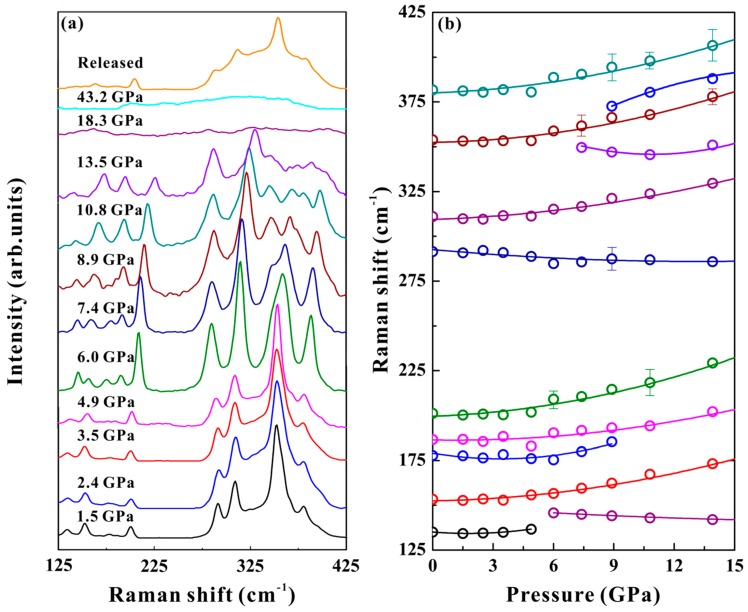
(**a**) Raman spectra of orpiment at selected pressures under hydrostatic condition (λ = 514 nm, T = 300 K). (**b**) Raman mode frequency evolution against pressure.

In this work, the Raman scattering experiments were conducted under non‒hydrostatic and hydrostatic conditions at a pressure range of 1 atm to 42.6 GPa. A series of non‒hydrostatic Raman scattering peaks and their corresponding Raman shift results are shown in Figure 3. Another similar hydrostatic Raman peak and its corresponding Raman shift data are given in Figure 4. As shown in Figure 3a, nine characteristic Raman active modes were observed at an ambient pressure, which were assigned as follows [29,30]. The peaks at 135 and 201 cm^−1^ stand for the As–S–As bending vibration. The peak at 153 cm^−1^ denotes to the As–As–S bending vibration. The peaks at 177 and 187 cm^−1^ were assigned to S–As–S bending vibrations. The peaks at 290 and 308 cm^−1^ denote antisymmetric As–S stretching vibrations. The peak at 353 cm^−1^ denotes the As–S stretching vibration. The peak at 380 cm^−1^ was assigned to the antisymmetric As–S–As stretching vibration. These obtained nine Raman vibration modes were consistent with the results reported by Cheng et al [29]. The Raman-active modes of orpiment continuously shifted towards higher frequencies with increasing pressure, except for the Raman mode with a peak position of 290 cm^−1^. When the pressure increased to above 6.5 GPa, some remarkable characteristics were detected in the Raman spectrum of orpiment. The modes with peak positions of 135 and 177 cm^−1^ merged with their neighboring modes at 6.5 and 9.1 GPa, respectively. Three new Raman-active modes appeared: A peak at 145 cm^−1^ at the pressure of 6.5 GPa; and peaks at 347 and 365 cm^−1^ at 7.6 GPa. The newly appeared Raman mode with a peak position of 145 cm^−1^ shifted to a lower frequency with increasing pressure, while the Raman modes with peak positions of 347 and 365 cm^−1^ shifted to higher frequencies. After the pressure was enhanced to 17.0 GPa, the Raman features tended to be difficult to distinguish, until they disappear. The Raman spectrum of orpiment upon decompression from 42.6 GPa recovered to the original state, which meant a reversible process under a non-hydrostatic condition.

The evolution of the experimental Raman modes in orpiment with increasing pressure is shown in Figure 3b. All of these Raman modes at ambient pressure showed nonlinear behavior with increasing pressure under our experimental conditions. The obtained fitting results and the pressure coefficients are summaries in Table 1, using the equation:

(1) $$\omega (P)=\omega_{0}+\alpha P+\beta P^{2}$$

where *ω*_0_ is the peak positions of the Raman modes at an ambient condition and *P* is the pressure. It was remarkable that several Raman vibration modes of orpiment exhibited moderate softening, merged with their neighbor modes and exhibited a complex splitting behavior in a given pressure range. The new appeared Raman modes above 6.0 GPa with peaks at 145,347 and 365 cm^−1^ were also detected by Mamandov et al. [31] at ambient pressure and a low temperature (*T* = 4 K). This phenomenon illustrated the complementary aspect of a low temperature (at *P* = 0) and a high pressure (at *T* = 300 K) for Raman spectroscopy: A low temperature reduced the line width to resolve as many structural features as possible for these complex spectra, while a high pressure revealed the structure by increased splitting at a constant line width [18]. All the obtained experimental points did not show obvious unusual behavior at any given pressure point. Indeed, the obtained relationship between the observed Raman modes and the pressure could be attributed to the variations of the atomic positions and bond lengths at high pressure. A similar trend existed for the hydrostatic conditions shown in Figure 4. Consequently, the results of Raman spectroscopy measurements at high pressure disclosed good phase stability of orpiment below 17.0 GPa.

**Figure 5:**
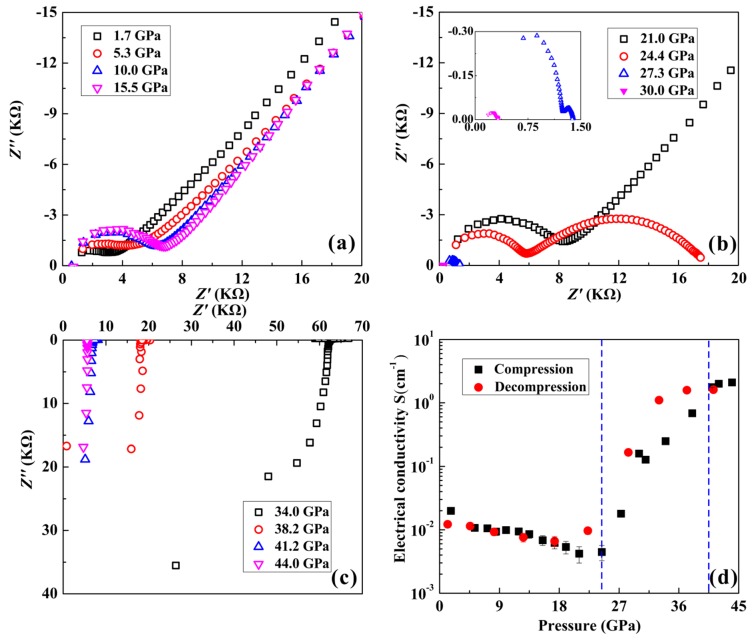
The electrical behaviors for orpiment with increasing pressure. (**a**–**c**) The complex impedance spectra of orpiment at selected pressures. (**d**) The variations of electrical conductivity for orpiment with the increasing pressure and decreasing pressure.

Both the variations in the distances of atoms and the structural phase transition in orpiment could tune its electronic properties. To further verify the phase transition and metallization of orpiment, electrical conductivity measurements were performed up to ~44.0 GPa at room temperature. The representative complex impedance spectra of orpiment under high pressure are shown in Figure 5a–c. The ZView software was used to fit the plots (equivalent circuit method). Two parts could be well identified in the frequency range 10^−1^–10^7^ of the impedance spectra: The semicircular arc in the higher frequency represented the resistance of the grain interior, whereas the oblique line at a lower frequency was characteristic of the grain boundary [32,33]. It is noteworthy that the resistance of grain boundary began to decrease above 24.4 GPa, and disappeared when the pressure was enhanced to 34.0 GPa. In this work, we paid more attention to the pressure effects of the grain interior contribution related to the phase transitions. The relationships between the electrical conductivity of orpiment and pressure in the process of compression and decompression at room temperature are shown in Figure 5d. As the pressure increased, the electrical conductivity of orpiment decreased before 24.4 GPa, and it then increased rapidly up to the pressure of 38.2 GPa. When the pressure was enhanced to 41.2 GPa, the electrical conductivity of orpiment remained relatively stable. The pressure-dependent electrical conductivity of orpiment in our experimental pressure range could be divided into three parts: An ambient pressure to 24.4 GPa with a rate of −0.020 S cm^−1^, 24.4 to 38.2 GPa with a rate of 0.162 S cm^−1^, and 38.2 to 44.0 GPa with a rate of 0.018 S cm^−1^. The variation in the conductivity of orpiment at 24.4 GPa was related to a pressure-induced phase transition. The increased overlap of the electronic orbital wave function and narrowing of the energy gap are reasons why the electrical conductivity of orpiment showed a rapidly increasing trend between 24.4 and 38.2 GPa (Figure 5d). Electrical conductivity of greater than 1 S cm^−1^ at a pressure above 38.2 GPa may be indicative of metallization. The electrical conductivity of orpiment was reversible upon decompression, which was consistent with the Raman spectroscopy measurements. This reversible phenomenon on the electrical conductivity of orpiment was different with Sb_2_S_3_, with a layer structure [12].

**Figure 6:**
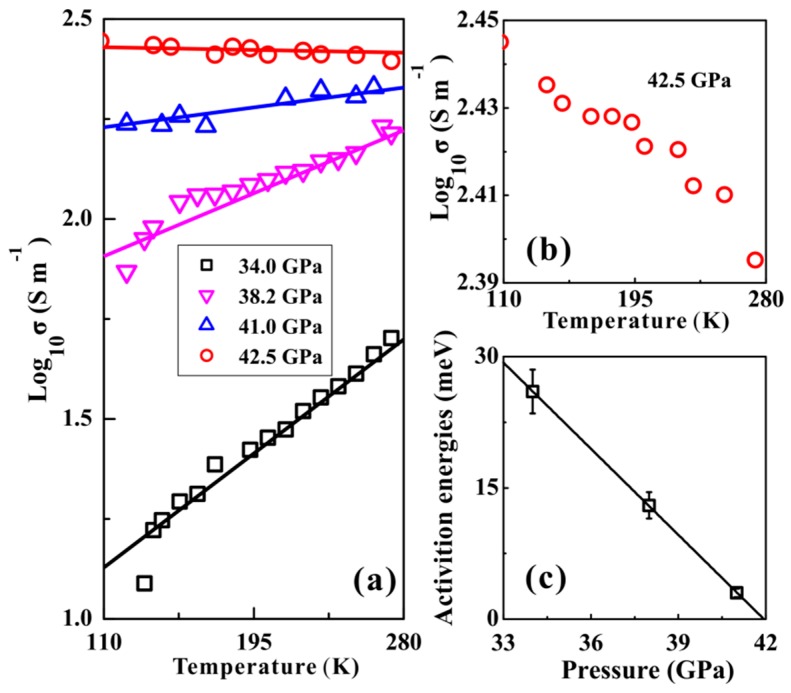
(**a**) The relationship between the temperature and electrical of orpiment at selected pressure. (**b**) Pressure dependence of activation energy for orpiment.

Temperature-dependent conductivity measurements of orpiment were performed to verify whether orpiment undergoes metallization. The results can be fitted by the Arrhenius equation. With increasing temperature, the electrical conductivity of orpiment increased below 41.0 GPa, which represented typical semiconductor behavior. It showed a negative relationship between the temperature and electrical conductivity at 42.5 GPa, which indicated a clear metallic behavior (Figure 6b). All of the obtained results revealed the occurrence of the semiconductor-metal transition of orpiment. According to the relationship between the temperature and electrical conductivity, the activation energy of orpiment at a selected pressure can be determined by:

(2) $$\sigma =\sigma_{0}\exp\left(-E_{t}/k_{b}T\right)$$

where *σ*_0_ stands for the pre-exponential factor (S cm^−1^), *E*_t_ stands for the activation energy (meV)—which could be determined by a linear fitting between the logarithmic conductivity and 1000/*T*—*k*_b_ stands for the Boltzmann constant, and *T* stands for the absolute temperature (K). The relationship between the activation energy of orpiment and pressure is shown in Figure 6c. The activation energy reduced with increasing pressure, which indicated that electrical transport of carriers became easier at high pressure. The pressure dependence of the activation energy can be fitted as a function of:

(3) $$E_{t}=-3.28+137.69P$$

where *E*_t_ denotes the activation energy and *P* denotes the pressure. The fitting results demonstrated that the value of *E*_t_ reached zero when the pressure was enhanced up to 41.9 GPa, as shown in Figure 6c. This meant that this sample became metal above 41.9 GPa, which was in good agreement with the electrical conductivity measured results.

**Figure 7:**
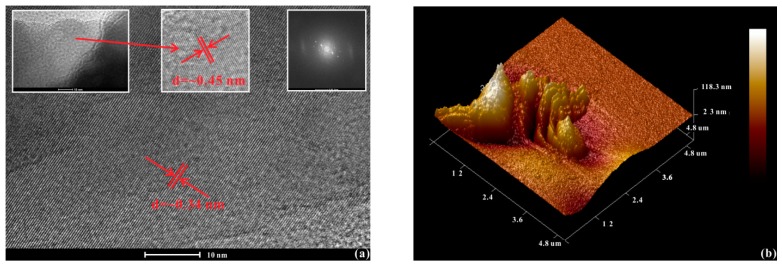
(**a**) The high-resolution transmission electron microscopy (HRTEM) images of decompressed orpiment from 44.0 GPa. Inset: the left one is a HRTEM image of the initial sample, the right one is a cross-sectional selected-area electron diffraction pattern by HRTEM for the decompressed orpiment. (**b**) The atomic force microscopy (AFM) image of orpiment upon decompression from 44.0 GPa.

To deeply explore the morphology and structural changes of orpiment after decompression from 44.0 GPa, the analysis for the recovered orpiment was performed by HRTEM and AFM (Figure 7). As shown in Figure 7a, the interlayer spacing of the starting material was ~0.45 nm. As for the recovered orpiment released from 44.0 GPa, it remained in the crystalline phase with an interlayer spacing of ~0.34 nm (Figure 7a). As shown in Figure 7b, there were crystal-shaped clusters on the recovered samples. Both the layer structure and surface morphology were well preserved upon decompression from 44.0 GPa. This reversible phenomenon was consistent with the results of the electrical conductivity and Raman spectroscopy measurements.

**Figure 8:**
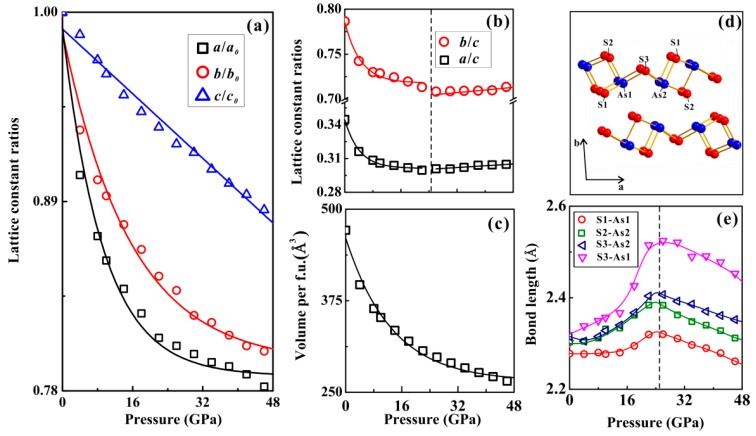
The variations of (**a**) lattice constant parameters (*a*/*a*_0_, *b*/*b*_0_ and *c*/*c*_0_), (**b**) axial ratios (*a*/*c* and *b*/*c*), (**c**) unit cell volume and bond length for orpiment at high pressure. Two regions could be determined by the black vertical dotted lines at the pressure of ~25 GPa (**d**) Crystalline structure of monoclinic (*P*2_1_*/c*) orpiment at ambient pressure. We also nominate the As and S ions in the crystalline structure. (**e**) Selected S-As bond lengths of orpiment up to 46 GPa.

To further investigate the phase stability of orpiment under high pressure, first-principles theoretical calculations were also performed at a pressure range from 0 GPa to 46.0 GPa. Some crucial crystalline parameters of orpiment, including the unit cell volume, lattice parameters, and bond angles were determined, as shown in Figure 8a–c. The obtained value of unit cell volume was 471.2 Å^3^ and the lattice parameters were *a* = 4.22 Å, *b* = 9.65 Å, *c* = 12.27 Å, and *β* = 109.59°, respectively, which was consistent with previously reported results [24,28]. The pressure dependence of the lattice parameters is shown in Figure 8a, from which the anisotropic compressibility of orpiment could be determined. In particular, the short *a* axis had better compressibility than the *b* and *c* axes, with increasing pressure. The stiffer lattice constant of the long *c* axis decreased with the increasing pressure, which showed a simple monotonic liner behavior with increasing pressure. By taking into account the lattice constant ratios, some interesting variations of pressure effects in both *a*/*c* and *b*/*c* were observed at ~25 GPa (Figure 8b). The compressibility of the initially softer *a* and *b* axes reach that of the *c* axis above ~25 GPa. The variations of axial compressibility were directly related to changes in interatomic parameters. There was a continuous change in the pressure dependence of the unit cell volume up to 46.0 GPa (Figure 8c). The pressure dependence of selected interatomic As–S bond lengths of orpiment at high pressure is shown in Figure 8e. The most significant stretching of the bond in the calculation below ~25 GPa, was the S3–As1 bond, while the S1–As1 bond showed the least significant stretching. For the S2–As2 and S3–As2 bonds, similar stretching was observed below ~25 GPa. The selected bond length showed the most obvious pressure-related effect, which underwent a reversal in the pressure dependence at ~25 GPa. The changes of the pressure effects in the bond lengths were linked to the compressibility changes of the axes, as mentioned above.

The type of phase transition can be determined by the characteristic parameter variations, such as the variations in the unit cell volume, lattice parameters, axial ratios, and bond lengths [22,23]. The collapse of a unit cell volume provides good evidence for the structural phase transition under high pressure. Whereas, the observed discontinuities in the axial ratios and bond lengths with the continuities in the unit cell volume and lattice parameter at a certain pressure are the characters for the isostructural phase transition. Thus, we attributed the variations in compressibility of orpiment at ~25.0 GPa to an isostructural phase transition.

**Figure 9:**
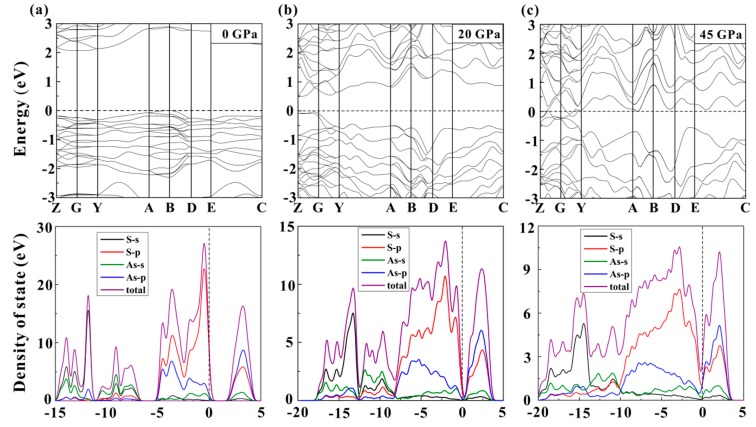
The selected band structure and the corresponding total and partial density for orpiment at different pressure. (**a**) and (**b**) The bandgap energy at 0 and 20 GPa are 2.07 and 0.51 eV, respectively. (**c**) The bandgap has already closed at the pressure of 45 GPa. The bandgap of orpiment narrows with increasing pressure.

At the same time, the evolution of the electronic properties of orpiment at high pressure was also revealed through first-principles theoretical calculations. The electrical structures of orpiment at different pressures are shown in Figure 9. These results predicted that orpiment had indirect bandgap energy of 2.07 eV under ambient conditions, which agreed well with a previous study [5]. The bandgap energy decreased to 0.51 eV at 20.0 GPa and then closed at 45.0 GPa, which indicated a clear metallicity of orpiment. These results provided more evidence for pressure-induced metallization of orpiment at around 42.0 GPa, which was consistent with the experimental results. As seen in Figure 9, the high-energy valence bands and the conduction band were controlled by the S–p and As–p states, respectively. The low-energy valence bands were contributed mostly by the S-s states, whereas, the middle-energy valence bands were contributed mostly by the As-s states. Electronic coupling and hybridization became gradually intense at a high pressure, which resulted in the broadening of the energy bands. Furthermore, compared with the conduction band, the high-energy valence band of orpiment showed a stronger broadening with increasing pressure, which could result in a decrease in the bandgap and even closing of the bandgap.

## 4. Conclusions

Using a DAC, we have found that the phase transition and metallization of orpiment occurred at about 25.0 and 42.0 GPa, respectively. The results were acquired by a combination of experimental and theoretical methods, including Raman scattering, impedance spectroscopy, electrical conductivity measurements at variable temperature, AFM, HRTEM, and first-principles calculations. The variable temperature electrical conductivity measurements and first-principles theoretical calculations provided strong evidence for a pressure-induced metallization at ~42.0 GPa. The images of the decompressed sample, from AFM and HRTEM, confirmed the well-preserved crystalline structure, which agreed well with the electrical conductivity and Raman spectroscopy measurements. Based on the observed compressibility change and the variation in pressure effects on the electrical conductivity, a second-order isostructural phase transition occurred at ~25.0 GPa. The observed high-pressure properties of orpiment will aid in the understanding of the universal crystal structure and electrical properties of *A*_2_*B*_3_-type materials. And furthermore, all of these high-pressure behaviors of orpiment will present some crucial information in the structural phase transition, metallization, amorphization and superconductivity for the *A*_2_*B*_3_-type of engineering materials at high pressure.

## Figures and Tables

**Table 1:** Relationship between the pressure and the Raman shift for orpiment as fitted with equation: $\omega (P)=\omega_{0}+\alpha P+\beta P^{2}$.

Mode Number	*ω*_0_ (cm^−1^)	*α* (cm^−1^GPa^−1^)	*β* (cm^−1^GPa^‒2^)
1	135	−0.420	0.120
2	153	0.240	0.095
3	177	−2.220	0.320
4	187	0.683	0.013
5	201	0.125	0.147
6	290	−0.891	0.046
7	308	−0.204	0.123
8	353	−0.193	0.118
9	380	0.474	0.107
